# Supplementary material for: HIF-PH Encoded by EGLN1 Is a Potential Therapeutic Target for Chronic Lymphocytic Leukemia
Source: Pharmaceuticals (Basel). 2022 Jun 10;15(6):734. doi: 10.3390/ph15060734 (PMC9229586; doi:10.3390/ph15060734)
Supplement: Supplementary file 1 [file pharmaceuticals-15-00734-s001.zip › pharmaceuticals-1729343-supplementary.pdf]

Supplement Table S1. Control scramble sequences and shRNA primer sequences for virus package.

ShRNA/scramble	Sequences (5′-3′)
shrna1	CCGGTGCACGACACCGGGAAGTTCACTCGAGTGAACTTCCCGGTGTCGTGCATTTTTG<br>AATTCAAAAATGCACGACACCGGGAAGTTCACTCGAGTGAACTTCCCGGTGTCGTGCA
shrna2	CCGGCTGTTATCTAGCTGAGTTCATCTCGAGATGAACTCAGCTAGATAACAGTTTTTG<br>AATTCAAAAACTGTTATCTAGCTGAGTTCATCTCGAGATGAACTCAGCTAGATAACAG
shrna3	CCGGGACGACCTGATACGCCACTGTCTCGAGACAGTGGCGTATCAGGTCGTCTTTTTG<br>AATTCAAAAAGACGACCTGATACGCCACTGTCTCGAGACAGTGGCGTATCAGGTCGTC
Scramble	CCGGCCTAAGGTTAAGTCGCCCTCGCTCGAGCGAGGGCGACTTAACCTTAGGTTTTTG<br>AATTCAAAAACCTAAGGTTAAGTCGCCCTCGCTCGAGCGAGGGCGACTTAACCTTAGG

Supplement Table S2. Specific sequences of the target gene primers.

primers	Sequences (5′-3′)
EGLN1	Forward: AGGCGATAAGATCACCTGGAT<br>Backward: TTCGTCCGGCCATTGATTTTG
CXCR4	Forward: GCAGCAGGTAGCAAAGTGAC<br>Backward: CCATGGTAACCGCTGGTTCT
HIF1A	Forward: GTCTGAGGGGACAGGAGGAT<br>Backward: CTCCTCAGGTGGCTTGTCAG
VEGF-A	Forward: AGGCCAGCACATAGGAGAGA<br>Backward: ACGCGAGTCTGTGTTTTTGC
TP53INP2	Forward: GCTGGTTTGTTACCCCTCCC<br>Backward: GGTGACGTAAACGGACATGCT
TP53I13	Forward: GAGCCCATTGTCCCGAGAG<br>Backward: GAGGGAAGGGTTAGCCATACA
TP53I3	Forward: GGAGGACCGGAAAACCTCTAC<br>Backward: CCTCAAGTCCCAAAATGTTGCT
TP53INP1	Forward: TTCCTCCAACCAAGAACCAGA<br>Backward: GCTCAGTAGGTGACTCTTCACT

NOL6	Forward: GGATCGGATTGATGCCTTCCT<br>Backward: GCTGCCCACAACAGTAACCT
WDR3	Forward: ACCAAGCAGTACCTACGCTAT<br>Backward:<br>TTCTCACCACGAAGTGTCACA
POP1	Forward: TTTTCAGACTCTGCCACGG<br>Backward: CGTCCGGTTCATGTGACATC
MDN1	Forward:<br>GGAATGCCGAAGCCATTAAAGC<br>Backward:<br>TTGCTCATCGACACACATAGC
